# Captopril reduces lung inflammation and accelerated senescence in response to thoracic radiation in mice

**DOI:** 10.1093/jrr/rraa142

**Published:** 2021-02-22

**Authors:** Ognoon Mungunsukh, Jeffy George, Elizabeth A McCart, Andrew L Snow, Joseph J Mattapallil, Steven R Mog, Ronald Allan M Panganiban, David L Bolduc, W Bradley Rittase, Roxane M Bouten, Regina M Day

**Affiliations:** Department of Pharmacology, Uniformed Services University of the Health Sciences, Bethesda, MD 20814, USA; Department of Pharmacology, Uniformed Services University of the Health Sciences, Bethesda, MD 20814, USA; Department of Pharmacology, Uniformed Services University of the Health Sciences, Bethesda, MD 20814, USA; Department of Pharmacology, Uniformed Services University of the Health Sciences, Bethesda, MD 20814, USA; Department of Microbiology, Uniformed Services University of the Health Sciences, Bethesda, MD 20814, USA; Center for Food Safety and Applied Nutrition, U.S. Food and Drug Administration, College Park, MD 20740, USA; Department of Pharmacology, Uniformed Services University of the Health Sciences, Bethesda, MD 20814, USA; Scientific Research Department, Armed Forces Radiobiology Research Institute, Uniformed Services University of the Health Sciences, Bethesda, MD 20889, USA; Department of Pharmacology, Uniformed Services University of the Health Sciences, Bethesda, MD 20814, USA; Department of Pharmacology, Uniformed Services University of the Health Sciences, Bethesda, MD 20814, USA; Department of Pharmacology, Uniformed Services University of the Health Sciences, Bethesda, MD 20814, USA

**Keywords:** angiotensin converting enzyme inhibitor, pulmonary fibrosis, ionizing radiation, mice, inflammation, total body irradiation, thoracic irradiation

## Abstract

The lung is sensitive to radiation and exhibits several phases of injury, with an initial phase of radiation-induced pneumonitis followed by delayed and irreversible fibrosis. The angiotensin-converting enzyme inhibitor captopril has been demonstrated to mitigate radiation lung injury and to improve survival in animal models of thoracic irradiation, but the mechanism remains poorly understood. Here we investigated the effect of captopril on early inflammatory events in the lung in female CBA/J mice exposed to thoracic X-ray irradiation of 17–17.9 Gy (0.5–0.745 Gy min^–1^). For whole-body + thoracic irradiation, mice were exposed to 7.5 Gy (0.6 Gy min^–1^) total-body ^60^Co irradiation and 9.5 Gy thoracic irradiation. Captopril was administered orally (110 mg kg^–1^ day^–1^) in the drinking water, initiated 4 h through to150 days post-irradiation. Captopril treatment increased survival from thoracic irradiation to 75% at 150 days compared with 0% survival in vehicle-treated animals. Survival was characterized by a significant decrease in radiation-induced pneumonitis and fibrosis. Investigation of early inflammatory events showed that captopril significantly attenuated macrophage accumulation and decreased the synthesis of radiation-induced interleukin-1β (IL-1β) and tumor necrosis factor-α (TNF-α) pro-inflammatory cytokines in the lungs of irradiated mice. Suppression of IL-1β and TNF-α correlated with an increase of the anti-inflammatory cytokine IL-10 in the spleen with captopril treatment. We also found that captopril decreased markers for radiation-induced accelerated senescence in the lung tissue. Our data suggest that suppression of inflammation and senescence markers, combined with an increase of anti-inflammatory factors, are a part of the mechanism for captopril-induced survival in thoracic irradiated mice.

## INTRODUCTION

The lung is uniquely sensitive to injury following radiation exposure. Injury to the lung has been observed following accidental radiation exposure and from clinical irradiation for the treatment of thoracic cancers. For patients undergoing radiation therapy for cancer treatment or bone marrow transplantation, lung injury is a significant limiting factor for the use of thoracic radiation therapy [1–4]. Delayed lung toxicity from radiation occurs in two phases, radiation-induced pneumonitis and fibrosis, both of which are significant causes of morbidity and mortality [5–7]. Although anti-inflammatory agents (steroidal and non-steroidal) are somewhat effective in treating low levels of inflammation associated with early radiation-induced pneumonitis [8], there are currently no agents approved by the Food and Drug Administration (FDA) for prevention or treatment of radiation lung fibrosis [9, 10].

Radiation-induced pneumonitis is characterized by increased levels of pro-inflammatory cytokines and inflammatory cells [4, 11–13]. The time courses of increased cytokines and inflammatory cells have been investigated in murine models of radiation-induced lung injury [4, 8, 12, 14, 15]. Several of the most studied cytokines—interleukin (IL)-1α, IL-1β, IL-6 and tumor necrosis factor-α (TNF-α)—display an early elevation within 1–24 h of radiation and a second phase of elevation at ~7 days [4, 12, 15]. These early transient increases are followed by sustained elevation starting at ~5 weeks post-irradiation [4, 12]. Cytokine production was associated with DNA damage, both within and outside of the radiation field, suggesting that heightened and prolonged inflammation contributes to cellular damage in the thoracic cavity even outside of the field of radiation [4]. The activation and accumulation of inflammatory cells in the lung tissues is an important feature of radiation-induced pneumonitis [4, 11, 13, 14, 16, 17]. Activated alveolar macrophages are the most abundant inflammatory cells noted during the progression of radiation-induced pneumonitis [18]. Inflammatory cells and cytokines contribute to alterations in other lung cells from ~3 months after radiation exposure, including alveolar epithelial cell and endothelial cell loss, inflammatory cell infiltration and alveolar hemorrhaging, leading to dyspnea with pulmonary edema [8, 14, 19].

Studies suggest that the late phase of pneumonitis overlaps with the initiation of radiation-induced fibrosis. Radiation-induced fibrotic remodeling is characterized by the irreversible loss of normal lung architecture through: (i) loss of normal alveolar epithelial and endothelial cells; (ii) appearance and proliferation of activated fibroblasts (myofibroblasts); and (iii) alteration of the extracellular matrix, especially the deposition of type I and III collagen [20]. Specific cytokines induced in the pneumonitis phase are believed to impact fibroblast growth and trans-differentiation to the myofibroblast phenotype. Increased expression of the pro-fibrotic cytokine transforming growth factor β1 (TGFβ1) displays a cyclic pattern similar to the pro-inflammatory cytokines, with early transient peaks between 1 and 24 h post-irradiation, a transient peak at ~7 days post-irradiation and a sustained increase starting ~4–7 weeks post-irradiation [4, 12]. Increased thickness of alveolar septa can be observed within 30 days of radiation, with increased collagen observable in alveoli within 11 weeks post-irradiation [21, 22]. An emerging view of radiation-induced fibrotic lung remodeling suggests that it may also evolve as the result of aberrant wound healing following pneumonitis, particularly involving apoptosis of parenchymal Type II alveolar epithelial cells and endothelial cells [23], and may be associated with senescence [14, 24].

The hormone angiotensin II (Ang II) is best known as a principal physiological regulator of blood pressure, blood volume and blood cell homeostasis. The production of Ang II is tightly regulated by its proteolytic activation from a pre-propeptide. Angiotensinogen is proteolytically cleaved to angiotensin I, which is further cleaved to the active form Ang II by angiotensin-converting enzyme (ACE) [25]. Remarkably, recent evidence suggests that Ang II can play a causative role in fibrosis of the lung and other tissues [26–29]. In the lung, Ang II has been implicated in the loss of normal epithelial and endothelial cells, and may also participate in the trans-differentiation and proliferation of myofibroblasts [29–32]. Local synthesis of Ang II has been observed in fibrotic plaques, and lung myofibroblasts obtained from human idiopathic pulmonary fibrosis tissue were found to generate Ang II [33–35]. Blockade of Ang II maturation by ACE inhibition was shown to reduce radiation injuries in a variety of tissues [36]. Treatment with ACE inhibitors and angiotensin type 1 receptor (AT1) antagonists mitigates pneumonitis and lung fibrosis in animal models induced by bleomycin [37] and by thoracic irradiation [38–40]. Inhibition of AT1 was shown to improve pulmonary function in patients with lung fibrosis in a retrospective clinical study [41]. The inhibition of ACE was also associated with reduced radiation-induced pulmonary toxicity in patients with thoracic cancers [42, 43].

Although the aforementioned studies have demonstrated that blockade of the Ang II pathway can mitigate radiation-induced lung injury, the mechanism behind this beneficial effect is unknown. Here we investigated the effect of the ACE inhibitor captopril on radiation-induced inflammation and cellular senescence in the lung at early and delayed time points post-irradiation to investigate radiation-induced events prior to the development of fibrosis. Our data demonstrate that captopril significantly reduced specific types of inflammation in the lung tissue following radiation injury and reduced radiation-induced accelerated senescence in lung cells.

## MATERIALS AND METHODS

### Reagents

Reagents were obtained from Sigma-Aldrich except where noted (Sigma-Aldrich, St Louis, MO, USA). Captopril (USP grade) was dissolved in acidified water at 0.55 g l^–1^. A previous study established the stability of captopril in acidified water [44].

### Animals

All experiments were conducted in compliance with the Animal Welfare Act, in accordance with the principles in the ‘Guide for the Care and Use of Laboratory Animals’, Institute of Laboratory Animal Resources, National Research Council, National Academy Press, 2011, and approved by the Institutional Animal Care and Use Committee. Female CBA/J mice were purchased from The Jackson Laboratories (Bar Harbor, ME, USA). This strain of mice has been shown to have radiation injuries that can be extrapolated to the human lung for evaluation of the mechanisms of action of radiation and for testing of countermeasures [45]. Mice were housed in groups of four in rooms maintained at 21 ± 2°C, 50 ± 10% humidity and a 12 h light/dark cycle in a facility accredited by the Association for Assessment and Accreditation of Laboratory Animal Care International. Commercial rodent ration (Harlan Teklad Rodent Diet 8604, Harlan Laboratories, Madison, WI, USA) and acidified water (pH = 2.5–3.0, to control opportunistic infections) were provided *ad libitum*.

### Thoracic irradiation and captopril treatment

Thoracic irradiations using the Philips Industrial 320 kVp machine (Model MG, Royal Philips, Amsterdam, The Netherlands) and the RS2000 Biological Research Irradiator (Rad Source Technologies, Suwanee, GA, USA) were performed using a custom 9 mm thick lead shield with thoracic cutouts and Lucite jigs [46]. The Philips irradiator was used for thoracic irradiations with 250 kVp and 12.0 mA filaments current, with the inherent beryllium filter, plus a 1.25 mm Cu and a 0.95 mm Al filter for beam hardening; the dose rate was 0.5 Gy min^–1^. The exposure dosage was calibrated using Lucite cylindrical phantoms (2.54 cm diameter × 7.62 cm length) located under the holes of the shield, each containing in its core three alanine dosimeters (Far West Technologies, San Diego, CA, USA). Doses to the alanine dosimeters were measured after simultaneous irradiation with an electron paramagnetic resonance spectrometer e-Scan (Bruker, Biospin, Billerica, MA, USA) to provide reproducibility of ≥0.5%. Due to decommissioning of the Philips Irradiator, the RS2000 Biological Research Irradiator was utilized for mechanistic studies. Irradiation was performed with the RS2000 using the following settings: 160 kVp, 25 mA, 90 s irradiation time and 0.3 mm Cu beam filtration; the dose rate was 0.749 Gy min^–1^. The approximate HVL provided by the manufacturer was 0.62 mm Cu. The University of Wisconsin Medical Radiation Research Center provided eight acrylic mouse phantoms with three (1 × 1 × 1 mm) Harshaw thermoluminescence dosimeter (TLD)-100 microcubes (Thermo Electron Corp., Oakwood Village, OH, USA) embedded in each phantom. The cylindrical phantom was 27 mm diameter × 65 mm length, stabilized by a cylindrical insert of 15 mm × 27 mm with a 3 mm thick stand. Dose measurements were conducted twice. TLD readouts were processed at the University of Wisconsin MRRC using a national standard with an expanded uncertainty (*k* = 2) of 5%. The dose rate was reported for each aperture position. The average absorbed dose rate for any position was 0.775 Gy min^–1^ with 97% uniformity. For thoracic irradiation, mice (12–14 weeks old) were anesthetized with intraperitoneal injections of 150 mg k^–1^ ketamine + 18 mg kg^–1^ xylazine. Anesthetized mice were secured with forelimbs out of the field in the prone position in Lucite jigs (3 mm thick). Thoracic X-rays confirmed positioning. Sham-irradiated (sham) mice underwent anesthesia and placement in jigs without irradiation. At 4 h post-irradiation, captopril was provided in acidified water, 0.55 g captopril l^–1^, to deliver 110 mg kg^–1^ day^–1^ per mouse based on water consumption calculated from previous experiments [47]. Groups for all experiments were sham + vehicle (acidified water), sham + captopril, thoracic irradiation + vehicle and thoracic irradiation + captopril.

### Dual thoracic and total-body irradiations

CBA mice were subjected to a split dose of radiation to determine captopril effects on hematopoietic and pulmonary injuries in the same animal. The initial total-body dose was 7.5 Gy (0.6 Gy min^–1^), followed by an ~30 min interval, but no more than 1 h, to allow for movement of mice from one apparatus to another. Mice were anesthetized for thoracic irradiation, as described above. The thoracic dose was 9.5 Gy, 0.6 Gy min^–1^, to equal a 17 Gy total thoracic exposure.

### Lung cell isolation and flow cytometry

Mice were euthanized and the lungs were perfused by injection of 10 ml of phosphate-buffered saline (PBS; Gibco Laboratories, Gaithersburg, MD, USA) through the right ventricle. The lungs were removed and rinsed in PBS, then transferred to a fresh tube containing RPMI and 10% fetal bovine serum (FBS), and kept on ice until dissociation. Single cells were isolated from lungs using the mouse lung dissociation kit (Miltenyi Biotec Inc., San Diego, CA, USA), a gentleMACS™ C tube and a gentleMACS™ dissociator according to the manufacturer’s instructions (Miltenyi Biotec Inc.). After lung dissociation, cells were passed through a 70 μm cell strainer and the remaining red blood cells were lysed using ACK lysis buffer (Lonza, Walkersville, MD, USA). The cells were washed twice using 10 ml of RPMI + 10% FBS. Cells were resuspended in ice-cold FACS buffer [1× PBS supplemented with 1% bovine serum albumin (BSA) and 0.1% NaN_3_] and counted using trypan blue exclusion dye. To block non-specific staining, lung cells were pre-incubated with rat anti-mouse CD16/32 antibody (clone 2.4G2, BD Biosciences, San Diego, CA, USA) for 30 min on ice followed by staining with saturating amounts of conjugated antibodies for 30 min. For the phenotypic analysis of T and B cells, cells were labeled simultaneously with CD45-PE (clone 30-F11), CD3-APC-Cy7 (clone 17A2), CD8-APC (clone 53-6.7), CD4-FITC (clone GK1.5) and B220 (clone RA3-6B2). Neutrophils, monocytes, macrophages and dendritic cells were discriminated based on the differential expression of CD45-PE (clone 30-F11), CD3-PE-Cy7 (clone 17A2), B220-PE-Cy7 (clone RA3-6B2), CD11b-APC-Cy7 (clone M1/70), Ly6G-PE-CF594 (clone 1A8), Ly6C-BV711 (clone HK1.4) and CD11c-FITC (HL3). All antibodies, except for Ly6C, in the second panel were obtained from BD Biosciences. Ly6C was obtained from Biolegend, Inc. (San Diego, CA, USA). All antibodies used in this study were titrated using mouse spleen cells.

**Fig. 1.**
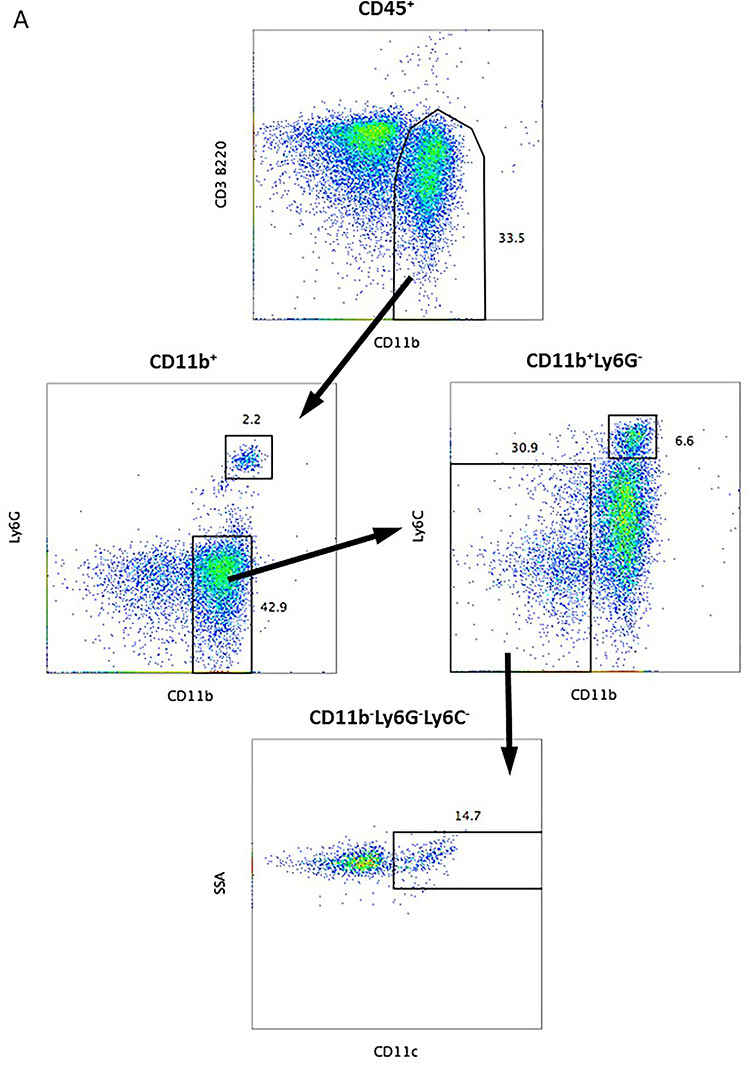
Gating strategy used to evaluate alveolar macrophages and dendritic cells in the mouse lungs. A sequential gating strategy was used to identify neutrophils (CD45^+^ CD3^–^B220^–^CD11b^+^Ly6G^+^), monocytes (CD45^+^CD3^–^B220^–^CD11b^+^Ly6C^+^) and alveolar macrophages and dendritic cells (CD45^+^CD3^–^B220^–^CD11b^–^Ly6G^–^Ly6C^–^CD11c^+^). Dead cells were excluded from the analysis using the live/dead marker VIVID.

All staining procedures were conducted on ice. Following staining, the cells were washed twice with ice-cold FACS buffer. The subsets studied included total T cells (CD45^+^CD3^+^), CD4^+^ T cells (CD45^+^CD3^+^CD4^+^CD8^−^), CD8^+^ T cells (CD45^+^CD3^+^CD8^+^CD4^−^) and B cells (CD45^+^CD3^−^B220^+^), neutrophils (CD45^+^CD3^−^B220^−^CD11b^+^Ly6G^+^), monocytes (CD45^+^CD3^−^B220^−^CD11b^+^Ly6C^+^), and alveolar macrophages and dendritic cells (CD45^+^CD3^−^B220^−^CD11b^−^Ly6G^−^Ly6C^−^CD11c^+^). Cells were labeled with live/dead amine reactive dye VIVID [48] to exclude dead cells. A total of 100 000 events were collected using a BD LSR II Cytometer (Becton Dickinson, BD, Franklin Lakes, NJ, USA). Flow cytometric data were analyzed using FlowJo Software Version 10.0 (Tree Star, Inc., Ashland, OR, USA), and the proportions of various subsets are presented as a frequency of total CD45^+^ cells. A sequential gating strategy was used to identify neutrophils (CD45^+^CD3^−^B220^−^CD11b^+^Ly6G^+^), monocytes (CD45^+^CD3^−^B220^−^CD11b^+^Ly6C^+^), and alveolar macrophages and dendritic cells (CD45^+^CD3^−^B220^−^CD11b^−^Ly6G^−^Ly6C^−^CD11c^+^). The gating strategy is shown in Fig. 1.

### RNA isolation

Lungs and spleens of mice were harvested after euthanasia. Small pieces were cut from spleens and all three lobes of the right lung (~30 mg total), transferred to a tube containing 0.4 ml of RTL buffer (Qiagen, Germantown, MD, USA) and homogenized with an Ultra Thorax homogenizer (Jahnke & Kunkel, Staufen, Germany). Tissue samples were homogenized again using QIAshredder mini columns (Qiagen) for 2 min at 16 000 *g*. RNA was isolated from the homogenate using the RNeasy mini kit (Qiagen) in accordance with the manufacturer’s instructions. Genomic DNA was removed using the RNase-free DNase Set (Qiagen). RNA was quantified spectroscopically (ND-1000 Spectrophotometer, NanoDrop Technologies, Wilmington, DE, USA), and RNA integrity was assessed by capillary electrophoresis (Bio-Rad Laboratories, Hercules, CA, USA).

### Preparation of cDNA and quantitative real-time PCR (qPCR)

Total RNA (1.0 mg) was reverse transcribed with the GeneAmp® RNA PCR kit according to the manufacturer’s protocol (Applied Biosystems, Foster City, CA, USA). A 5 μl aliquot of cDNA (diluted 1:10) was subjected to a 20 μl qPCR. The following primer pairs were used: TNF-α, 5′-TCTTCTCATTCCTGCTTGTGG and 5′-GGTCTGGGCCATAGAACTGA; IL-10, 5′-CAGAGCCACATGCTCCTAGA and 5′-TGTCCAGCTGGTCCTTTGTT; IL1-β, 5′-TGAGCACCTTCTTTTCCTTCA and 5′-TTGTCTAATGGGAACGTCACAC; Gro-1a, 5′-GACTCCAGCCACACTCCAAC and 5′-TGACAGCGCAGCTCATTG; and CDKN1A (p21), 5′-TCCACAGCGATATCCAGACA and 5′-GGACATCACCAGGATTGGAC. qPCR was performed in triplicate reactions containing 12 μM of each primer pair and 10 μl of 2× iTaq™ Universal SYBR® Green Supermix (Bio-Rad) and using an iCycler with a IQ5 optical system (Bio-Rad Laboratories). Absence of non-specific amplification was confirmed by analysis of qPCR products using agarose gel electrophoresis. As internal control, the mRNA level of α-tubulin was determined using the primer pairs 5′-CTCCATCCTCACCACCCACAC-3′ and 5′-CAGGGTCACATTTCACCATCT-3′. For quantification, the comparative threshold cycle (CT) method (or 2^–ΔΔCT^) was used to assess relative changes in mRNA levels between the vehicle-treated control and the drug-treated and/or irradiated animal samples [49].

### Lung histology

Tracheotomies were performed on euthanized mice, and custom-built cannulas were inserted. Lung lavage was done using 1.0 ml of saline. Bronchoalveolar lavage (BAL) fluid was centrifuged at 360 *g* for 10 min at 4°C to collect cells and the supernatant was stored at −80°C until further analysis. The lungs were perfused with PBS by injection of the left heart ventricle. Lungs were then inflated and perfused with formalin through the cannula in the trachea and fixed for 2 days in formalin. Formalin-fixed and embedded lung sections were cut and stained with either hematoxylin–eosin (H&E) or Masson’s trichrome. In the mouse lung sections stained with H&E and Masson’s, the pathologist (S.R.M.) used a semi-quantitative scoring system for two histopathology parameters, chronic pneumonitis (inflammation) and fibrosis. The latter parameter was scored based on the collagen stain (Masson’s trichrome). The severity scoring of each parameter was a modified Ashcroft pulmonary fibrosis score 0–5 (normal/no lesion = 0, minimal = 1, mild = 2, moderate = 3, marked = 4 and severe = 5) [50, 51]. In this study, the highest and most severe scores for pneumonitis and fibrosis in an irradiated mouse lung were 4 and 3, respectively.

### Western blotting

Lung tissue was harvested after euthanasia and all three lobes of the right lung were transferred to a fresh tube and stored immediately at −80°C. To prepare whole-cell lysates, 0.5 ml of RIPA buffer [(50 mM Tris, pH 8.0; 150 mM NaCl, 0.5% sodium deoxycholate, 0.1% SDS) containing protease and phosphatase inhibitor cocktails (Roche Diagnostics GmbH, Mannheim, Germany), supplemented with 1 mM phenylmethylsulfonyl fluoride (PMSF)] was added to the lung sample, homogenized using a Dounce homogenizer and incubated for 1 h at 4°C. Samples were cleared by centrifugation at 14 000 *g* at 4°C. Protein concentrations were determined by BCA Protein Assay (Thermo Fisher Scientific Inc., Waltham, MA, USA). Samples (50 mg of protein) were separated by SDS–gel electrophoresis and then electroblotted onto a polyvinylidene difluoride (PVDF) or nitrocellulose membrane. Membranes were blocked with 5% blocking reagent (BioRad Laboratories Inc.) or with 50% LI-COR blocking buffer (Lincoln, NE, USA) diluted with Tris-buffered saline (TBS). The following primary antibodies were used for immunoblotting: anti-β-actin (sc-47778, 1:1000, Santa Cruz Biotechnology Inc., Santa Cruz, CA, USA) and anti-p21/waf1 (sc-397; Santa Cruz Biotechnology), anti-β-actin (A1978, 1:5000; Sigma-Aldrich) and anti-caspase-3 (#9665, 1:1000; Cell Signaling Technologies, Danvers, MA, USA). Proteins were detected with horseradish peroxidase-linked secondary antibodies (1:5000) and SuperSignal West Pico (or Dura) Chemiluminescent Substrate (Pierce, Rockford, IL, USA). Alternatively, secondary antibodies were used from LI-COR: IRDye 680 goat anti-rabbit IgG or IRDye 800 goat anti-mouse IgG (LI-COR). Proteins were detected using an Odyssey CLx (LY-COR). WCIF ImageJ software (http://www.uhnresearch.ca/facilities/wcif/index.htm) was used for densitometry analysis.

### Statistical analysis

Statistical analysis was performed using the non-parametric *t*-test and one-way analysis of variance (ANOVA) and the Sidak test with SPSS Statistics, Version 22 software and GraphPad Prism Version 6 (GraphPad Prism Software, Inc. San Diego, CA, USA). Significance was considered at *P* < 0.05. Error bars represent the standard error.

## RESULTS

### Captopril treatment increased survival in CBA mice exposed to thoracic X-irradiation and inhibits the development of pulmonary fibrosis

**Fig. 2.**
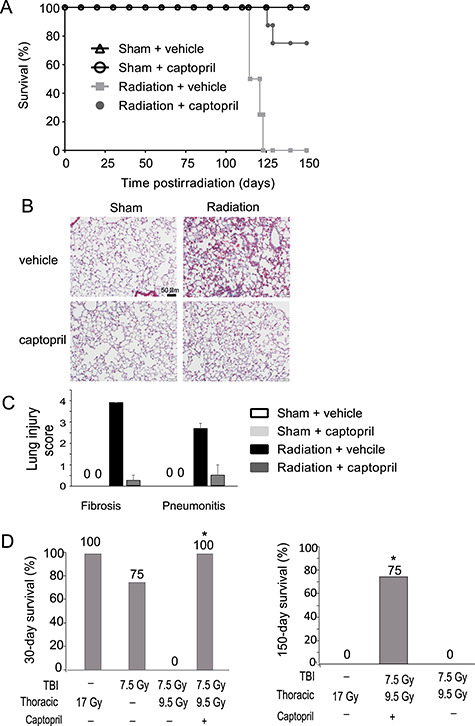
Captopril improves survival from thoracic and total-body irradiation (TBI) and mitigates radiation-induced pneumonitis and fibrosis. (A–C) CBA mice (12–14 weeks of age) were either sham irradiated (anesthetized, no irradiation) or exposed to 17 Gy (0.5 Gy min^–1^) thoracic irradiation. Mice were treated with either vehicle or captopril. (A) Kaplan–Meier survival curve up to 150 days post-irradiation. *n* = 8/group. (B) Masson’s trichrome stain of lung tissue at 110 days post-irradiation. Representative sections are shown from *n* = 4/group. (C) Pneumonitis and fibrosis scores of lung tissue at 110 days post-irradiation. Scores ranged from 0 to 5 for both pneumonitis and fibrosis. Data shown are means ± SD (*n* = 4 animals). (D) CBA mice (12–14 weeks of age) were exposed to either 17 Gy (0.6 Gy min^–1^) thoracic irradiation, 7.5 Gy (0.6 Gy min^–1^) TBI or 7.5 Gy TBI + 9.5 Gy thoracic irradiation. Left panel: survival at 30 days post-irradiation; right panel: survival at 150 days post-irradiation. ^*^Significantly different from the untreated, irradiated group. *n* = 8/group.

We investigated the effects of captopril on survival of CBA mice exposed to thoracic irradiation. In vehicle-treated mice, exposure to 17 Gy thoracic irradiation resulted in 0% survival by 125 days post-irradiation, with mortality between 110 and 120 days post-irradiation (Fig. 2A). In contrast, captopril treatment, initiated 4 h post-irradiation and maintained through day 150 post-irradiation, resulted in 75% survival at 150 days post-irradiation. Lung tissue was obtained for histology from sham-irradiated and 17 Gy thoracic-irradiated animals, both with and without captopril treatment at 110 days post-irradiation (Fig. 2B, C). Histological sections were scored by a veterinary pathologist blinded to the treatment groups. Treatment with captopril resulted in no histologically observable alterations in lung architecture in sham-irradiated animals, and all sham-irradiated animals exhibited scores of 0 for fibrosis and pneumonitis. In contrast, exposure to 17 Gy thoracic irradiation induced marked fibrosis (score 4 ± 0 SD) and pneumonitis (score 2.75 ± 0.25 SD) in the mice as compared with sham-irradiated groups within 110 days post-irradiation. Captopril treatment reduced irradiation-induced fibrotic remodeling (0.25 ± 0.25 SD) and pneumonitis (0.5 ± 0.5 SD) in animals exposed to thoracic irradiation.

It was previously demonstrated that captopril treatment post-irradiation improved survival in mice exposed to total-body irradiation through the protection of specific hematopoietic cell lineages [47, 52, 53]. To determine the effects of captopril on hematopoietic and pulmonary radiation injuries in the same animal model, we investigated the effects of captopril treatment on animals exposed to both total-body and thoracic irradiation. Previous studies indicated that 7.5 Gy total-body irradiation induced ~LD_50/30_ hematopoietic injury in CBA mice. Mice were exposed to an additional 9.5 Gy thoracic irradiation to give a total thoracic exposure of 17 Gy. Interestingly, exposure to an LD_50/30_ dose of total-body irradiation plus thoracic irradiation resulted in 100% mortality within 30 days, suggesting that thoracic irradiation increased the toxicity of the total-body irradiation (Fig. 2D). Captopril treatment resulted in 100% survival at 30 days, suggesting that captopril mitigated radiation-induced hematopoietic injuries in the dual exposure. Captopril treatment also resulted in 75% survival from the total-body + thoracic irradiation at 150 days, compared with 0% survival for animals exposed to either 17 Gy thoracic irradiation alone or dual exposure (Fig. 2D). These data confirm that captopril can protect both the bone marrow and lung in an animal model with both types of radiation injuries.

### Captopril prevents radiation-induced increase of alveolar macrophage levels

**Fig. 3.**
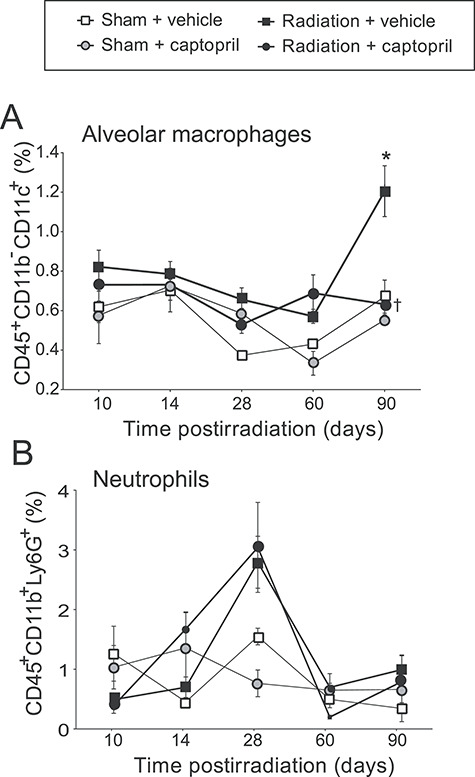
Captopril reduces the radiation-induced increased alveolar macrophages at 90 days post-irradiation but not early increases in neutrophils. CBA mice (12–14 weeks of age) were either sham irradiated (control) or exposed to 17.9 Gy (0.749 Gy min^–1^) thoracic irradiation. Mice were treated with either vehicle or captopril. Isolated cells were labeled with a panel of markers to discriminate alveolar macrophages (plus dendritic cells) (A) or neutrophils (B) as described in the Materials and Methods, and are shown as a percentage of CD45^+^ leukocytes. Data shown are means ± SEM, *n* = 6/group. ^*^Significantly different from sham + vehicle (control) (*P* < 0.05). †Significantly different from radiation + vehicle (*OP* < 0.05).

We investigated the effect of captopril on early inflammation by examining an early time course of changes in lung-derived alveolar macrophage, neutrophil and lymphocyte numbers in CBA mice exposed to 17.9 Gy thoracic irradiation. Cell subsets were identified by flow cytometry as described in the gating strategy (see Fig. 1). Captopril treatment of non-irradiated animals did not result in any detectable increase in alveolar macrophages or neutrophils compared with vehicle-treated non-irradiated animals (Fig. 3). (Note that both alveolar macrophages and dendritic cells are both positive for the CD11c marker. Our histological examination revealed that most of the immune cells in the lungs are alveolar macrophages.) At 90 days post-irradiation, the levels of alveolar macrophages increased significantly in vehicle-treated, irradiated mice (Fig. 3A, *P* < 0.05). Captopril suppressed alveolar macrophage accumulation in the lungs at this time point (*P* < 0.05). Neutrophils displayed a trend toward increased levels on day 28 post-irradiation in both vehicle-treated and captopril-treated irradiated animals compared with sham-irradiated groups, but this did not reach significance (Fig. 3B). We did not observe significant differences in the numbers of CD4^+^ or CD8^+^ T cells, or B cells in lungs of the different groups (data not shown).

### Radiation-induced expression of pro-inflammatory cytokines is suppressed by captopril

**Fig. 4.**
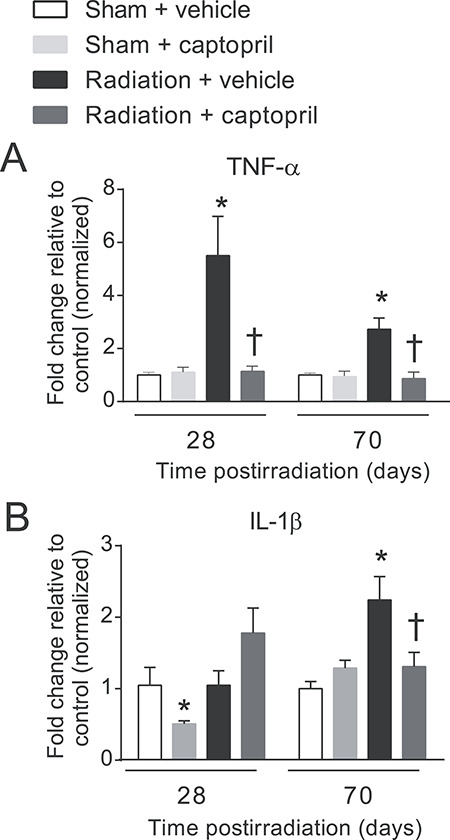
Captopril reduces radiation-induced inflammatory cytokine production in the lung. CBA mice (12–14 weeks of age) were either sham irradiated (control) or exposed to 17.9 Gy (0.749 Gy min^–1^) thoracic irradiation. Mice were treated with either vehicle or captopril. Lung tissue was obtained at the indicated time points and mRNA was used for qRT-PCR for TNF-α (A) or IL-1β (B). Data shown are means ± SEM, *n* = 6/group. ^*^Significantly different from sham + vehicle (control) (*P* < 0.05). †Significantly different from vehicle-treated, irradiated mice (*P* < 0.05).

TNF-α and IL-1β pro-inflammatory cytokines are elevated in the lung at early time points following radiation exposure, and are believed to contribute to the development of radiation-induced pneumonitis and fibrosis [4, 12, 15]. We investigated the effects of captopril on radiation-induced expression of TNF-α and IL-1β in the lung following 17.9 Gy thoracic irradiation. TNF-α expression was significantly increased in the irradiated vehicle-treated animals at days 28 and 70 post-irradiation [~6-fold and ~ 2.5-fold, respectively (*P* < 0.05)] compared with sham animals (Fig. 4A). While captopril treatment had no effect on TNF-α expression in sham-irradiated animals, the increase of TNF-α expression by radiation was significantly suppressed by captopril treatment at 28 and 70 days post-irradiation, to near baseline levels (*P* < 0.05). IL-1β expression was significantly elevated (~2-fold, *P* < 0.05) by radiation 70 days post-irradiation in vehicle-treated animals (Fig. 4B). Captopril treatment significantly reduced radiation-induced IL-1β expression at this time point (*P* < 0.05). Interestingly, captopril also reduced basal levels of IL-1β in sham-irradiated animals at 28 days post-irradiation (Fig. 4B).

### Expression of the anti-inflammatory cytokine IL-10 in the lung and spleen is enhanced by captopril after radiation

**Fig. 5.**
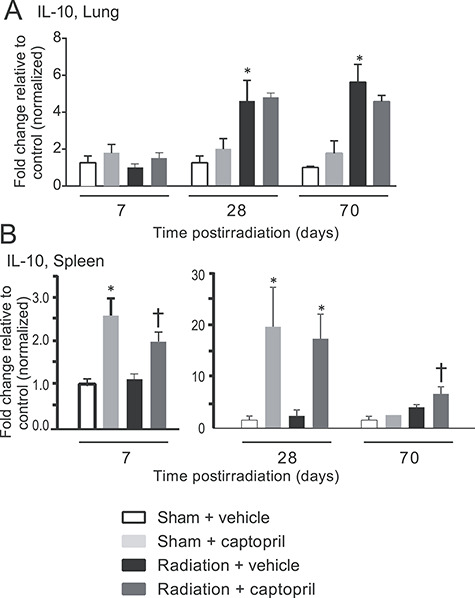
Radiation induces local IL-10 expression in the lung, but captopril treatment induces IL-10 expression in the spleen. CBA mice (12–14 weeks of age) were either sham irradiated (control) or exposed to 17.9 Gy (0.749 Gy min^–1^) thoracic irradiation. Mice were treated with either vehicle or captopril. Lung (A) and spleen (B) tissues were obtained at the indicated time points and mRNA was used for qRT-PCR for IL-10 gene expression. Data shown are means ± SEM, *n* = 6/group. ^*^Significantly different from sham + vehicle (control) (*P* < 0.05). †Significantly different from treated, irradiated animals (p < 0.05).

IL-10 is an immunosuppressive cytokine, demonstrated to regulate the expression of TNF-α and IL-1β [54, 55]. IL-10 expression has been suggested to be a mechanism for the reduction of fibrotic remodeling in a carbon tetrachloride (CCl_4_) model for liver injury and in a murine model of chronic renal disease [56, 57]. We therefore investigated localized IL-10 expression in the lungs of non-irradiated and irradiated mice. Radiation significantly induced IL-10 expression in the lungs. At days 28 and 70 post-irradiation, IL-10 expression in vehicle-treated irradiated animals was increased by ~ 5-fold at both times compared with sham control animals (Fig. 5A). The radiation-induced IL-10 expression in the lungs was not further increased by the captopril treatment (Fig. 5A). Captopril also did not show any significant effect on IL-10 expression in non-irradiated (sham) animals (Fig. 5A).

The spleen has previously been reported to be a significant source of IL-10 for the suppression of systemic inflammation [58, 59]. We therefore also investigated captopril regulation of IL-10 in the spleens of non-irradiated and irradiated mice. Interestingly, thoracic irradiation had no effect on IL-10 expression in the spleen (Fig. 5B). However, captopril treatment induced the expression of IL-10 in both groups of mice. In non-irradiated animals, captopril induced an ~2.5-fold and ~17-fold increase in IL-10 after 7 and 28 days, respectively, compared with vehicle-treated animals (*P* < 0.05) (Fig. 5B). IL-10 expression in the captopril-treated non-irradiated animals returned to basal levels by day 70. In the spleens of irradiated animals, IL-10 was increased by treatment with captopril by 25-, 20- and 6-fold on days 7, 28 and 70 post-irradiation, respectively, compared with vehicle-treated irradiated animals (*P* < 0.05) (Fig. 5B).

### Captopril reduces expression of markers for senescence post-irradiation in lung tissue *in vivo*

**Fig. 6.**
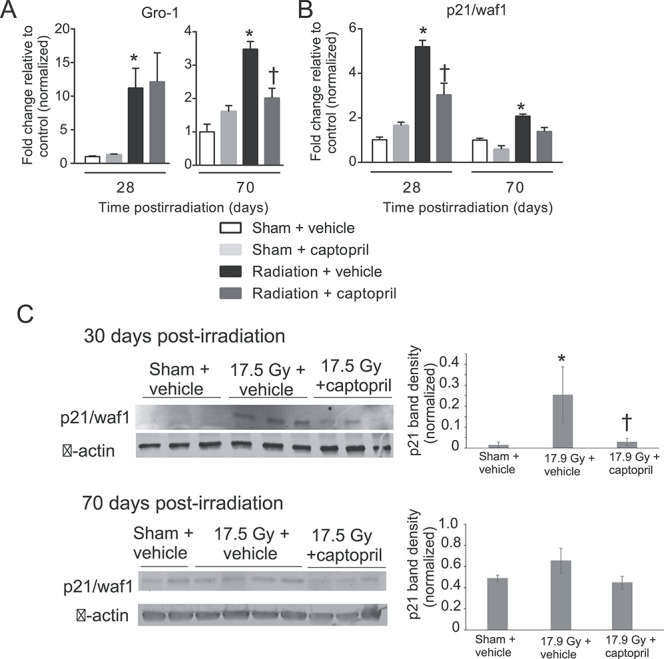
Captopril reduces radiation-induced senescence but not radiation-induced apoptosis in the lung. CBA mice (12–14 weeks of age) were either sham irradiated or exposed to 17.9 Gy (0.749 Gy min^–1^) thoracic irradiation. Mice were treated with either vehicle or captopril. Lung tissue was obtained at the indicated time points. (A, B) qRT-PCR was performed using lung tissue mRNA for Gro-1 (A) or p21/waf1 (B). Data shown are means ± SEM. ^*^Significantly different from sham + vehicle (control) (*P* < 0.05, *n* = 6/group). †Significantly different from vehicle-treated, irradiated animals (*P* < 0.05, *n* = 6/group). (C) Western blots were performed for p21/waf using lung lysates at the indicated time points post-irradiation. Blots were subsequently probed for β-actin as a loading control. Representative blots are shown. Bar graphs show densitometric analysis of western blots normalized to actin levels. For 30 days post-irradiation *n* = 3/group. For 70 days post-irradiation: sham + vehicle *n* = 6; 17.9 Gy + vehicle *n* = 8; 17.9 Gy + captopril *n* = 7. Data shown are means ± SEM. ^*^Significantly different from control (*P* < 0.05). †Significantly different from vehicle-treated, irradiated animals (*P* < 0.05).

Senescence has been shown to occur in the lung following radiation exposure, and may be associated with inflammation and fibrosis [60]. We investigated the effect of captopril on radiation-induced senescence by examining mRNA expression of growth-regulated oncogene 1 (Gro-1), a potent inducer of senescence [61], and of the cell cycle checkpoint protein p21/waf at days 28 and 70 post-irradiation (Fig. 6). Thoracic irradiation induced Gro-1 ~12-fold at 28 days post-irradiation and ~3.5-fold at 70 days post-irradiation (Fig. 6A). Captopril treatment significantly reduced Gro-1 expression on day 70 post-irradiation (*P* < 0.05). The p21/waf1 gene expression was induced by radiation exposure ~5.5-fold at day 28 post-irradiation, and ~2-fold at day 70 post-irradiation (Fig. 6B). Captopril significantly prevented the radiation-induced increase of p21/waf1 at day 28 post-irradiation (*P* < 0.05). Western blots for p21/waf demonstrated that radiation up-regulates this protein at 30 days post-irradiation, and captopril significantly reduced this up-regulation (Fig. 6C, upper panel). A trend toward increased p21/waf was also observed at 70 days post-irradiation (Fig. 6C, lower panel). Interestingly, radiation also activated caspase-3, a marker of apoptosis, at both time points, but captopril did not reduce caspase-3 activation (data not shown).

## DISCUSSION

Exposure to high levels of ionizing radiation in humans results in severe and usually fatal multiorgan damage. Although countermeasures are currently available for acute hematopoietic injuries by radiation, definitive FDA-approved post-irradiation countermeasures are lacking for other tissues, especially for the lung. ACE inhibitors have been shown to improve survival in rodents exposed to thoracic irradiation. Additionally, clinical trials of patients receiving thoracic irradiation for cancer treatment have demonstrated benefits for the use of ACE inhibitors [29, 37, 40]. However, the mechanism of ACE inhibition for protection against radiation-induced lung injury is not known, and this knowledge is required for FDA approval of radiation countermeasures under the Animal Rule [62]. Here we have demonstrated that treatment with captopril can enhance survival in a model of radiation lung injury from thoracic irradiation as well as a model of hematopoietic and lung injury from total-body + thoracic irradiation. It was recently demonstrated that survival from hematopoietic acute radiation syndrome by captopril is associated with the suppression of radiation-induced acute inflammatory response [47]. Our current data demonstrate that captopril-induced survival from thoracic irradiation is also associated with suppression of radiation-induced pulmonary inflammation.

Radiation-induced pneumonitis is associated with recruitment and activation of inflammatory cells. Chiang *et al.* reported finding a significant increase of lung neutrophils following thoracic irradiation [13], and several studies reported increased numbers of alveolar macrophages following thoracic irradiation in rodents [4, 63, 64]. Our study used flow cytometric analysis of total lung cells to demonstrate that both neutrophils and alveolar macrophages are increased following radiation exposure. Although the increase in neutrophils was not affected by captopril treatment, the radiation-induced increase in alveolar macrophage levels was significantly attenuated by captopril treatment. Because the time course of activation of alveolar macrophages is closely correlated with both radiation-induced pneumonitis and fibrosis [64], we hypothesize that suppression of the radiation-induced increase in alveolar macrophages is a critical effect of captopril for the mitigation of lung toxicity by radiation.

The elevation of pro-inflammatory cells in the lung is associated with increased local expression of cytokines and chemokines post-irradiation [4, 13]. IL-1β and TNF-α are produced by activated macrophages and are associated with airway hyper-responsiveness and pneumonitis [65, 66]. Additionally, TNF-α expression is believed to be important for subsequent fibrosis following radiation exposure [13]. Our data demonstrate that captopril suppressed TNF-α and IL-1β expression, which correlated with the decreased levels of alveolar macrophages. The suppression of these cytokines is consistent with the reduced levels of alveolar macrophages by captopril treatment post-irradiation. Our recently published report demonstrated that captopril suppressed the expression of acute inflammatory response proteins, including serum amyloid A protein (SAA), in a murine model of hematopoietic acute radiation syndrome following total-body irradiation [47]. Interestingly, we did not identify increased SAA levels in the serum of mice exposed to thoracic irradiation (data not shown), suggesting that an acute inflammatory response may occur only following total-body irradiation and that thoracic irradiation is not sufficient to induce this response. The mechanism by which captopril exerts anti-inflammatory activity is not known. Previous studies have indicated that captopril has significant antioxidant activity *in vitro*, and that captopril treatment up-regulates copper/zinc-superoxide dismutase and non-enzymatic antioxidant activity in some tissues by 17–54% [67, 68]. However, several studies using antioxidants as radiation countermeasures for radiation-induced lung fibrosis have had mixed results, suggesting that antioxidant therapy alone may not be sufficient to prevent radiation lung injuries [69–72]. Our current data suggest that captopril treatment modulates a variety of pro-inflammatory responses as well as senescence from radiation.

A previous study of thoracic irradiation in rats demonstrated that treatment with captopril, enalapril (another ACE inhibitor) and an angiotensin receptor blocker (L 158,809) was sufficient to block the production of TGF-β1, a primary cytokine implicated in the development of pulmonary fibrosis [39]. However, the mechanism(s) by which blockade of the Ang II signaling pathway attenuates TGF-β1 after radiation remained unknown. Anti-inflammatory cytokines have been demonstrated to modulate inflammation and attenuate fibrotic remodeling in several animal models of fibrotic organ diseases. As an example of this, Louis *et al.* used the CCl_4_ model for the induction of liver fibrosis to demonstrate that endogenous IL-10 expression reduced levels of TNF-α and TGF-β, and prevented neutrophilic infiltration of the liver and liver fibrosis through decreased local collagen production [73]. Our data show that IL-10 was locally up-regulated in the lung in response to radiation, but that this was not significantly affected by captopril. In contrast, captopril treatment induced the expression of IL-10 in the spleen in both sham-irradiated and thoracic-irradiated animals. The splenic increase in IL-10 by captopril may reflect a general state of immunomodulation, with distal effects on radiation-induced inflammation in the lung. Further investigation is necessary to understand the anti-inflammatory effects of captopril and their mechanism(s).

Local inflammation can also be affected by senescent cells in tissues, and these cells have been demonstrated to actively secrete pro-inflammatory cytokines [74]. Several reports have demonstrated that accelerated senescence is a primary response of normal (non-transformed, non-immortalized) endothelial cells to radiation in culture [75, 76]. Senescence is also believed to contribute to reduced repair and fibrotic remodeling in the lung following radiation exposure [24, 77–79]. We investigated two markers of senescence in the lung tissue following thoracic irradiation to determine whether senescence might also be regulated by captopril treatment. Our data indicate that markers of senescence are up-regulated in lung tissue following thoracic irradiation, and that captopril treatment mitigated their expression at different time points. Suppression of senescence may also contribute to the reduction of pro-inflammatory cytokines in the lung following thoracic irradiation, but the specific contribution of these cells to radiation pneumonitis and fibrosis requires further investigation.

Interestingly, a previous study of thoracic irradiation in the rat by van der Veen *et al*. (2015) found that ACE inhibition attenuated cardiopulmonary injury following thoracic irradiation [80]. This study showed the benefit of ACE inhibition for the prevention of early (8 weeks) radiation-induced changes in breathing rates, early fibrotic changes and alveolar inflammation primarily when the heart was included in the radiation field. The data suggested that the effect of ACE inhibition reduced radiation-induced lung injury by preventing acute cardiac damage by radiation. Our studies include the heart in the radiation field, raising the possibility that cardiac damage is a comorbidity in our study.

In conclusion, we demonstrated that improved survival with captopril treatment after thoracic irradiation is associated with the suppression of radiation-induced increases in alveolar macrophages and pro-inflammatory cytokine synthesis. The suppression of alveolar macrophage proliferation and reduced inflammatory cytokine expression correlated with increased expression of the anti-inflammatory cytokine IL-10 in the spleen, as well as decreased expression of senescence markers in the lung. These studies involved a single dose of captopril, and further studies could investigate lower doses of captopril administration; a recent publication demonstrated that lower doses of captopril were effective for improving survival and hematopoietic recovery following thoracic irradiation, suggesting that reduced doses of the ACE inhibitor may also be effective [47]. In summation, our data provide the first mechanistic insights to explain the beneficial effects of Ang II blockade in mitigating radiation-induced lung injury.
